# High Color Purity Plasmonic Color Filter by One-Dimensional Photonic Crystals

**DOI:** 10.3390/nano12101694

**Published:** 2022-05-16

**Authors:** Jun Yong Kim, Hyo Jong Cho, Yun Seon Do

**Affiliations:** School of Electronic and Electrical Engineering, Kyungpook National University, 80, Daehak-ro, Daegu 41566, Korea; rhawns4567@knu.ac.kr (J.Y.K.); chj5611@knu.ac.kr (H.J.C.)

**Keywords:** nanostructure, surface plasmonics, photonic crystal, color filter

## Abstract

Structural colors have been reported instead of conventional dye- or pigment-based color filters. Color selectivity can degrade as structure-based optical resonances are accompanied by several resonance modes. In this work, we suggest a simple and effective design of the plasmonic color filter (PCF) that integrated the PCF with the one-dimensional (1D) photonic crystal (PhC). The introduced PhC creates an optical band gap and suppresses undesired peaks of the PCF caused by the high-order resonance mode. Finally, the suggested structure provides a high color purity. This study can be a guideline for technology that replaces conventional color filters.

## 1. Introduction

Electrically reproduced scenes with color are more familiar with a user’s experienced real world, leading to high accessibility of electrical data. Similarly, chromatic functions play a key role in the use of digital devices by providing meaningful information. Beyond modulating digital information, there have been recent widening efforts to use it by merging the real environment as augmented reality for education, medicine, military, and entertainment. Using the real situation and artificial digital information, color reproduction is important for providing natural visual experiences. Conventional chromatic components in electrical devices are based on organic or inorganic color-resist materials, such as pigments or dyes. These material-based colors exhibit large degradation from heat, light, and chemicals, originating from the material stability. Their general fabrication process, such as photolithography technology, has limits in producing a pattern in the subwavelength dimension [1].

Alternatively, nanostructure-based colors (‘structural colors’) have been reported. They represent colors by accompanying optical resonances [2,3,4,5,6,7,8,9,10]. Considering that structural colors do not possess coloring materials, they exhibit superior durability to conventional colors from external environmental factors. In particular, metal nanostructures can confine a large amount of light in a tiny volume within the optical frequency range. They enhance surface plasmons (SPs); therefore, collectively oscillating movements of the free electrons in metal at the dielectric–metal interfaces can be observed [11]. In addition, the material composition containing metal can be used as an electrical component because of its high conductivity. Therefore, plasmonic structures can be fabricated for novel devices performing multiplicative functions, to render the device structure thin, simple, flexible, and transparent.

Consequently, we report PCFs based on two-dimensional (2D) hole arrays in the thin metal film as follows: a design method for practical applications [12]; fabrication process for the mass production [13]; novel multifunctional application [14]. The SP resonance enhances the transmittance through the nanoholes at the resonance frequency (or wavelength) and finally results in selective spectral transmission. The 2D isotropic periodicity makes the SP resonance independent of the polarization state of the incident light. However, the higher-order resonance modes corresponding to the reciprocal vectors of the periodicity exhibit multiple transmittance peaks. These modes can hinder spectral selectivity and degrade the color purity.

Based on previous studies, here we suggest a design method for PCF with 2D nanohole arrays for enhancing color purity. The transmission spectra of the suggested structure were controlled using several additional layers. The optimized design from numerical simulations was fabricated. The optical effects of the modified structure were theoretically interpreted by measuring the performances of the calculated and fabricated structures.

## 2. Experimental Setup

To generate SP resonance, aluminum (Al) and lithium fluoride (LiF) were used. The high plasma frequency of the metal and the small value of permittivity of the dielectric reduces the resonance wavelength (*λ_sp(m,n)_*), as derived by Equation (1) [15],

(1) $$\lambda_{sp\left(m,n\right)}=\frac{P}{\sqrt{m^{2}+n^{2}}}\sqrt{\frac{\varepsilon_{m}\varepsilon_{d}}{\varepsilon_{m}+\varepsilon_{d}}}$$

where *P* is the period of the hole arrays with (*m,n*) numbers corresponding to the reciprocal vectors of the periodic orders of the square array, and *ε_m_* and *ε_d_* are the permittivity of the metal and dielectric, respectively. When the target wavelength was determined, the small value of the material part in Equation (1) resulted in a large dimension of *P* and secured more fabrication margin.

Calculations were performed using the finite domain time difference method (FDTD Solutions, Lumerical Co., Vancouver, BC, Canada). A unit cell with a single hole was modeled for a periodic domain under symmetry and anti-symmetry boundary conditions in x and y directions, respectively. For the z-direction, a perfect matching layer was used. A plane wave source within a wavelength range of 400–800 nm was propagated from the bottom in the z direction. The optical constants of Al, LiF, and tungsten oxide (WO_3_) were referred from the experimental data, and Palik data were used for the other materials [16]. The conventional dye-based color filters, which are patterned by photo-lithography, have limits for making smaller pixels because of the diffraction limit. In this point of view, color filters based on nanostructures can be good alternatives for realizing tiny pixels. Color filter pixels in micrometer scale included in the imaging devices such as CMOS image sensors or displays are affected by the normal incident light responses dominantly. Therefore, we investigated the optical characteristics of the normal transmission in each case.

The optimized structure was fabricated on the glass substrate. The dielectric materials and Al were deposited by thermal evaporation. Laser interference lithography was used to induce periodicity. Coherent two beams that were split from one light source of a frequency-doubled argon-ion laser produced a 1D periodic intensity profile. In a negative photoresist layer, the two-beam interference pattern was exposed twice in a perpendicular direction to each exposure. Details are described elsewhere [13].

Finally, the performance of the fabricated filter was measured. The optical transmittance was measured with a spectrophotometer (UV-2550, Shimadzu, Kyoto, Japan). The color gamut was checked using a luminance colorimeter (BM7-A, TOPCON, Tokyo, Japan), and an image was obtained using a microscope.

## 3. Results and Discussion

The first order of SP resonance occurring at the lowest energy, i.e., the longest wavelength region, produces the strongest interaction between the SPs and incident light and results in high transmittance [13]. Therefore, the transmissive type of the plasmonic filters based on periodic domains use the first-order resonance as the pass-band. To use the imaging device, the color filter should possess a single selective pass-band in primary colors, (red, green, and blue).

Considering that the peak of resonance wavelengths appears in a short wavelength region, as expected in Equation (1), the effects of the multiple resonance orders have to be verified, except for the blue filter. Equation (1) provides less information, such as the effect of scattering, which could be affected by the size, shape of holes, and grating depth. However, it provides exact relative comparability between the *λ_sp(m,n)_* corresponding multi-resonance orders. As expected from Equation (1), the second resonance order mode [*λ_sp(1,1)_*] appears within the wavelength range $1/\sqrt{2\;}$ times, compared with that in the first-order mode [*λ_sp(1,0)_* or *λ_sp(0,1)_*]. The red filters, for example, exhibit the main transmittance peak within the range of 600–700 nm, and the other transmittance peak originated from the second-order resonance roughly within the range of 424–495 nm. The additional transmission in the blue regent degrades the color purity, but the undesired transmission is inevitable.

**Figure 1:**
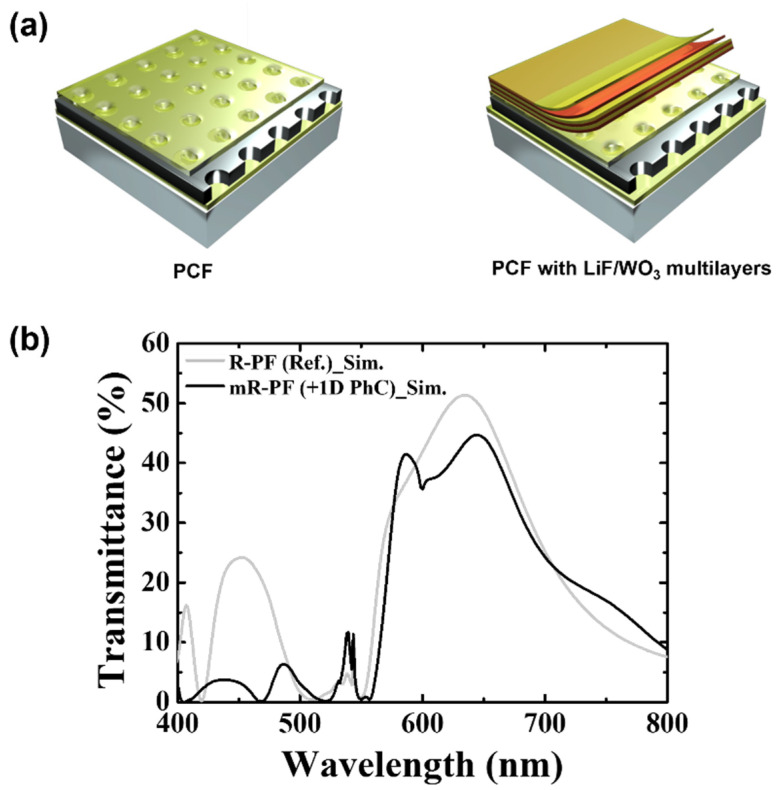
(**a**) Schematic diagrams of the R-PF as a reference (left) and the suggested mR-PF with multilayers; (**b**) simulated transmission spectra of the R-PF and mR-PF.

To quantitatively analyze the enhancement of the color performance, the optical response of the plasmonic red filter (R-PF) for the reference and modified red filter (mR-PF) was numerically simulated. Figure 1a illustrates the structure of R-PF. On a glass substrate, a 50 nm thick LiF layer, 150 nm thick Al layer, and a 150 nm thick LiF were sequentially stacked. The Al layer was perforated with 240 nm holes, which were separated with 370 nm of the period in the horizontal and vertical directions. The spectral characteristic of the R-PF is plotted in the graph, as shown in Figure 1b, with a gray line. The transmission spectrum shows 51.4% of the maximum transmittance at 634 nm with 131 nm of the full width at half maximum (FWHM). The additional peak of the second-longest wavelength region [with the maximum value at *λ_sp(1,1)_*] results in 24.2% of the maximum value at 452 nm. The color of the light in this range is extremely far different from red. Additionally, the ratio of the average of the undesirable transmittance (420–512 nm) to the main peak transmittance (547–780 nm) is 36.3%.

We propose an optical design to suppress the transmittance peaks originating from the higher-order resonance modes by importing a photonic band gap (PBG). When two materials with different refractive indexes from each other are infinitely repeated, the structure [PhC] induced a PBG in the direction of the repetition [17,18]. For the lights in the PBG, photonic modes cannot be allowed in the structure. The light with the frequency at the edge of the PBG is strongly confined in the PhC, the lights within the PBG are reflected from the PhC, and the lights in the other range travel through the PhC. We suggest the introduction of a PBG effect to the reference using the 1D PhC, which comprised a finite number of layers as few as possible. The designed PBG exists around the *λ_sp(1,1)_* of the R-PF to suppress the undesired transmission. Additionally, the mR-PF does not exhibit degradation in the pass-band.

The top of the R-PF is modified with multilayers, as illustrated in Figure 1a. The repetition of optical constants correlates to the top surface of the R-PF; therefore, the mR-PF exhibits the PBG effects through the transmission path. The additional layers comprise LiF and WO_3_.

The imported PhC is not infinitely stacked; therefore, it reveals an imperfect band gap; 100% reflection or confinement is not expected. The strength of the PBG effect and bandwidth of the PBG is affected by the ratio of refractive indices between two materials. First, LiF was used as the continuum of the R-PF to reduce loss during the incidence of the PhC from the R-PF. In addition, the refractive index of LiF (~1.39) is small, similar to those of transparent dielectric materials. To form a PBG with as few layers as possible, a high contrast ratio of refractive indices is required. Materials that can be handled by thermal evaporation are advantageous, considering the in situ fabrication process. Therefore, WO_3_ with 2.0–2.2 of refractive index in the visible range was used.

The center wavelength of PBG ($\lambda_{PBG\_C})\;$is related to the optical length affected by the refractive index (*n*) and thickness of each layer (*d*) as follows [19]:

(2) $$\lambda_{PBG\_C}=4nd$$

Because of the imperfect PBG, the transmission spectrum of the PhC has a deep valley around $\lambda_{PBG\_C}$ and additional shallow ripples, which originated from the interference between the reflected light modes from the LiF and WO_3_ interfaces, as shown in the Appendix A and supplementary information (Figure S1). To enhance the color purity of the R-PF, the multilayer stack was designed to form a deep and wide valley range near 450 nm corresponding to the *λ_sp(1,1)_* of R-PF. In addition, only one peak is required between 600 and 700 nm to maintain the maximum transmittance in the main pass-band region [around *λ_sp(1,0)_*]. Using Equation (2), the thicknesses of LiF and WO_3_ were set to 80 and 53 nm, respectively. Three pairs of WO_3_ and LiF were used. This is because when the PhCs were three pairs, their transmittance spectra exhibit low transmission at the *λ_sp(1,1)_* while maintaining high transmission at the *λ_sp(1,0)_* of R-PF (Figure S1).

The modified transmission characteristics of the mR-PF produced by introducing three pairs of LiF and WO_3_ are examined, as shown in Figure 1b, with the black line. Compared with the reference, the transmittance spectrum of the mR-PF exhibits several differences, such as a long-wavelength shift, suppressed undesired peak, and inflection points. The mR-PF exhibits distortion under the dominant trends, which is expected as the effect of the multilayer. The main peak shape is distorted with 10 nm of the peak shift to the right side and 10 nm of a wide FWHM. The maximum transmittance value was reduced by 6.7%p. The intensity of the undesired peak (second-order peak) decreased significantly by 17.5%p. In addition, the ratio of the average undesirable transmittance (420–512 nm) to the main peak transmittance (547–780 nm) was reduced by 27.7%p from 36.3% (R-PF) to 8.6% (mR-PF). The suppressed undesired peak improves the filtering properties. However, the shape of the transmittance is distorted due to the creation of a valley around 600 nm. This will be covered in greater detail later.

The redshift of the main peak can be understood using the dielectric media change at the upper interface. To apply the modification in the upper dielectric structure (in addition to the 1D PhC pairs and finite thickness of LiF), the dielectric constant of Equation (1) was modified from the bulk medium values to the effective refractive index, $n_{eff}$, obtained from a previous study [20,21,22].

(3) $$n_{d\_eff}=\sqrt{n_{1}{}^{2}x+n_{2}{}^{2}\left(1-x\right)}$$

Equation (3) indicates the effective refractive index of two stacked structures; $n_{1}$ and $n_{2}$ are the bulk refractive indices, respectively; *x* is the volume occupied by $n_{1}$ in the stacked structure.

(4) $$n_{eff}=n_{media}+\left(n_{d\_eff}-n_{media}\right)\left(1-e^{-2t/\delta_{d\_eff}}\right)$$

**Figure 2:**
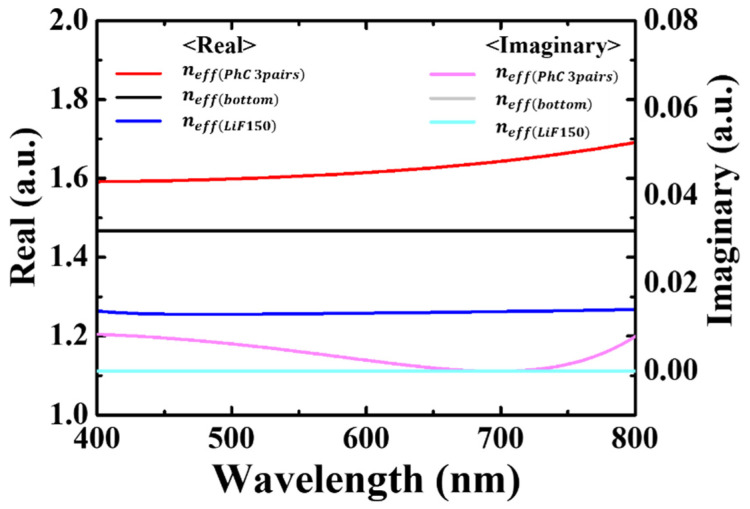
Refractive index of each dielectric structure: 150 nm thickness of LiF with 3 pairs of 1D PhC, 150 nm thickness of LiF, and bulk LiF.

Equation (4) is an equation for the effective refractive index in the plasmonic structure; $n_{media}$ and $n_{d\_eff}$ are the refractive indices of the surrounding media (air in this case) and the thin deposited layer on the metal surface, respectively; t is the thickness of the deposited layer; and $\delta_{d\_eff}$ is the decay length of the SP in a continuous dielectric film. To calculate the final effective refractive index of the mR-PF at the upper interface, the results obtained from Equation (3) were used in Equation (4). Figure 2 shows the bulk refractive index and effective refractive indices calculated using the aforementioned equations. The imaginary part of all refractive indices is close to zero in the visible range. For the real part, the effective refractive index of a finite thick LiF (150 nm), $n_{eff\left(LiF150\right)}$, is approximately 1.25–1.26, and that of the bottom side (LiF/Glass) is approximately 1.47, respectively. $n_{eff}$ is a refractive index of a structure with three pairs of (LiF/WO_3_) PhC atop the LiF (150 nm), calculated using Equations (3) and (4).

For the mR-PCF, the calculated $n_{eff\left(PhC\;3pair\right)}\;$resulted in the real part of 1.59–1.69, which is 0.34–0.43 higher than the $n_{eff\left(LiF150\right)}.\;$The mR-PF has a higher effective refractive index of the upper dielectric of the filter than that of the R-PF; therefore, it can be expected that the peak wavelength (*λ_sp_*) had shifted a long-wavelength through Equation (1). However, this is not proportional to the peak wavelength exactly to the ratio of the refractive index of the filters to the upper dielectric. This is because Equation (1) does not consider the diffraction/interference effect on the size of the hole. Therefore, it may be used to determine only the peak wavelength shift.

**Figure 3:**
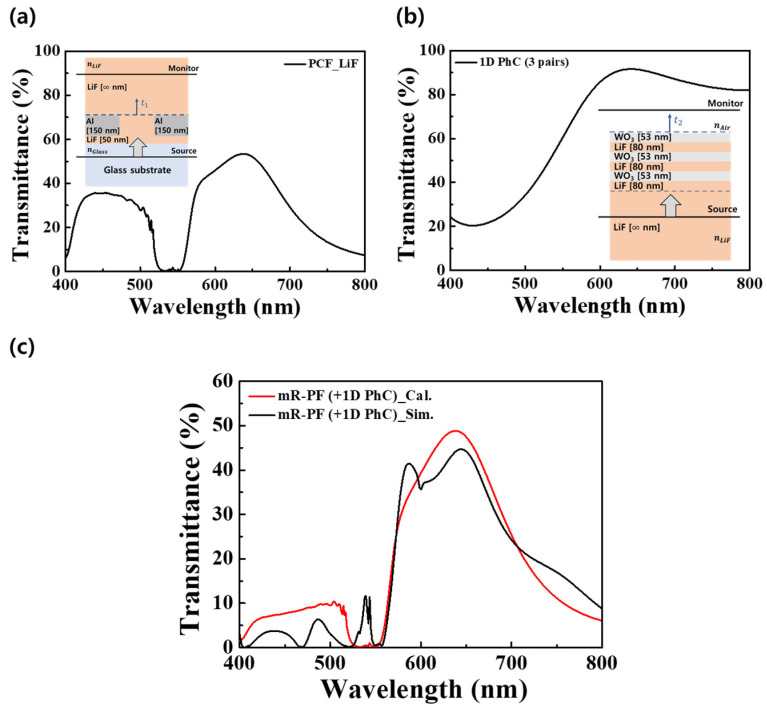
Optical response of mR-PF. Transmission spectra of (**a**) the light transmitted from the glass substrate through to the LiF layer via the PCF structure (inset: schematic of the PCF structure used in the FDTD simulation) and (**b**) the light transmitted from the LiF layer to the air via the 1D PhCs (inset: schematic of the 1D PhC structure used in the FDTD simulation). (**c**) Calculated and simulated transmittance for the mR-PF.

To understand the mechanism of the mR-PF, we analyze the optical characteristics of the PCF and 1D PhC separately. Figure 3 shows the calculated and simulated spectra for mR-PF. In determining the spectral properties of the PCF with mR-PF, the optical effects of the PCF and 1D PhC are predominant. To validate the optical effects of PCF and 1D PhC, we confirm the transmissive spectra of the optical structures inserted in Figure 3a,b using the FDTD simulation. The total transmittance (*T_total_*) of the mR-RF can be simply modeled as the product of the transmittances of the PCF (*T_PCF_*) and 1D PhC (*T_PhCs_*) [23].

(5) $$T_{Total}=T_{PCF}\times T_{1D\;PhC}=\left(\frac{n_{LiF}}{n_{Glass}}\times {\left|t_{1}\right|}^{2}\right)\times \left(\frac{n_{Air}}{n_{LiF}}\times {\left|t_{2}\right|}^{2}\right)$$

where $n_{Glass}$, $n_{LiF}$, and $n_{Air}$ are the refractive indices of the glass, LiF, and air, respectively, and $t_{1}$ and $t_{2}$ are the transmission coefficients of light transmitted through the PCF and 1D PhC structures. Figure 3a shows the calculated transmission spectrum of the PCF structure. The *T_PCF_* is a quantitative value of the light transmitted from the glass substrate through to the LiF layer via the PCF structure. This spectrum exhibited a major peak at a wavelength of 638 nm and an undesirable passband in the range of 398–531 nm. The main peak was 53.4%, and the average value of a passband from 398–531 nm was calculated to be 27%. Figure 3b shows the calculated spectrum of a structure, which is light transmitted from the LiF layer to the air layer via the 1D PhC. Through the PBG effect of 1D PhC, the transmission spectrum had a deep valley near $\lambda_{PBG\_C}$ (445 nm). Figure 3c shows the calculated and simulated spectra of the mR-PF. Using Equation (5), we calculate the *T_Total_* using the *T_PCF_* and *T_1D PhC_.* In the calculated transmission spectrum, the value of the main peak at 638 nm was 48.8%, and the average value of an undesired passband from 398–531 nm was 6.8%. Owing to the PBG effect of 1D PhC, the average of the unnecessary passband decreased by approximately 41.4%, compared with the R-PF. In addition, the main peak shifted to a longer wavelength by approximately 4 nm. Although the simulated and calculated spectra of the mR-PF are similar, there are differences at specific wavelengths. It indicates that additional optical effects are required to accurately predict the transmission spectrum of the mR-PF.

**Figure 4:**
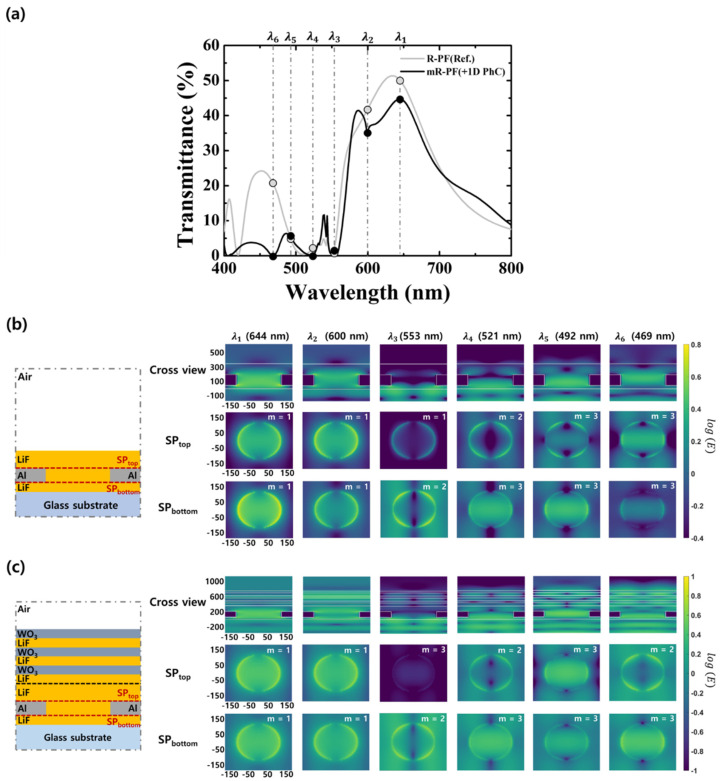
Spectral response and profile images of the electric field of the R-PF and mR-PF. (**a**) Transmission spectra of the R-PF and mR-PF at inflection points [$\lambda_{1}$ (644 nm), $\lambda_{2}$ (600 nm), $\lambda_{3}$ (553 nm), $\lambda_{4}$ (521 nm), $\lambda_{5}$ (492 nm), and $\lambda_{6}$ (469 nm)]. Schematic of the simulation structures and electric field profile images for (**b**) the R-PF and (**c**) mR-PF at the inflection points.

To estimate the valley from the simulation results, we use the FDTD simulation to confirm the electric field profile images at inflection points in the transmission spectrum of the mR-PF. Figure 4 shows the spectral response and electric field profile images of the R-PF and mR-PF. Figure 4a shows the inflection points in the transmission spectra of the R-PF and mR-PF, as shown in Figure 1b. The inflection points appear in the wavelength range of $\lambda_{1}$ (644 nm) to $\lambda_{6}$ (469 nm). To verify the variation in the SP modes of the PCFs by the 1D PhC, we analyze profile images of the electric field at the inflection points. Figure 4b,c illustrate the profile images of the electric field for the R-PF and mR-PF at the inflection points. There are two distinct modes in the PCF structure: those originating from the top metal–dielectric interface (SP_top_) and those originating from the bottom metal–dielectric interface (SP_bottom_).

The profile images of the electric field for the R-PF are shown in Figure 4b. As the wavelength decreased, the SP_top_ and SP_bottom_ sequentially increased into first-, second-, and third-order resonant modes (m = 1, 2, and 3) for the R-PF. When the SP_top_ and SP_bottom_ were in first- and third-order resonant modes simultaneously, the light incident on the glass substrate was transmitted into the air through the PCF at the following inflection points: $\lambda_{1}$ (644 nm), $\lambda_{2}$ (600 nm), $\lambda_{5}$ (492 nm), and $\lambda_{6}$ (469 nm). The electric field was simultaneously excited around the rims of the nanohole at the SP_top_ and SP_bottom_, resulting in resonance within the nanohole when the polarization direction of the electric field of the incident light was the same as that of the excited electric field at the nanohole. This contributes to the transmissive energy of the R-PF. At an inflection point of $\lambda_{1}$ (644 nm), high transmittance can be achieved in the matching modes of SP_top_ and SP_bottom_. When the second-order resonant mode (m = 2) was generated around SP_top_ and SP_bottom_, the transmittance was low at the following inflection wavelengths: $\lambda_{3}$ (553 nm) and $\lambda_{4}$ (521 nm). The electric field was concentrated on the upper or lower Al and LiF interfaces in the second-order resonant mode. This electric field decreases in the z-axis direction by the SP penetration depth and does not contribute to the transmissive energy.

Figure 4c illustrates the electric field profile images for the mR-PF. Dissimilar to the R-PF, the SP_top_ of the mR-PF exhibited discontinuity with the first-, third-, second-, third-, and second-order resonant modes because of the effect of the 1D PhC as the wavelength decreased. When the inflection points were $\lambda_{3}$ (553 nm), $\lambda_{4}$ (521 nm) and $\lambda_{6}$ (469 nm), the resonant modes of the SP_top_ were the second-order mode. As previously mentioned, transmittance was low at certain inflection points, including the second-order resonant mode of the SP_top_ or SP_bottom_. Compared with the R-PF, the valley at $\lambda_{6}$ (469 nm) was caused by the second-order resonant mode of the SP_top_. Additionally, the valley at $\lambda_{2}$ (600 nm) occurred because of trapped light within the 1D PhC. It indicates that the 1D PhC in the mR-RF can reduce the transmittance at undesirable wavelength bands as well as the variation of the SP_top_ resonant mode. Therefore, the 1D PhC can suppress unwanted peaks of the PCF and create inflection points by changing the SP mode of the PCF.

**Figure 5:**
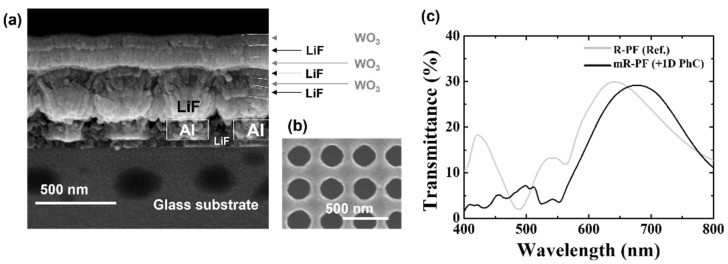
(**a**) Scanning electronic microscopy (SEM) image of the PCF containing 6 layers of PhC, (**b**) SEM image of the photoresist layer after laser interference lithography, (**c**) transmittance spectra of the R-PF and mR-PF.

Finally, each filter was fabricated, and the optical characteristics are examined as shown in Figure 5. After evaporating LiF and Al in turn, periodic holes were created by laser interference lithography. Figure 5b shows the holes on the photoresist layer after developing. Thereafter, the Al layer was etched by reactive ion etching and evaporated several LiF and WO_3_ layers, as designed. Details of the process are described elsewhere [13]. Figure 5c shows the transmittance properties of each fabricated structure. The position of the main peak shifted a long-wavelength from 643 (R-PF) to 678 nm (mR-PF). The intensity of the peak decreased by 0.73%p from 29.96 (R-PF) to 29.23% (mR-PF). The spectra were redshifted by the addition of the 1D PhC structure. Furthermore, it indicates that undesired additional peaks in the short wavelength region of the R-PF significantly decrease. As shown in Figure 5a, the reason for the difference from the simulation results is that the surface morphology is not flat. The optical properties of the PhC are most affected by the periodicity of each thick layer. If the surface morphology is not flat, the thickness of the layers varies, resulting in mismatched periodicity and faults.

**Figure 6:**
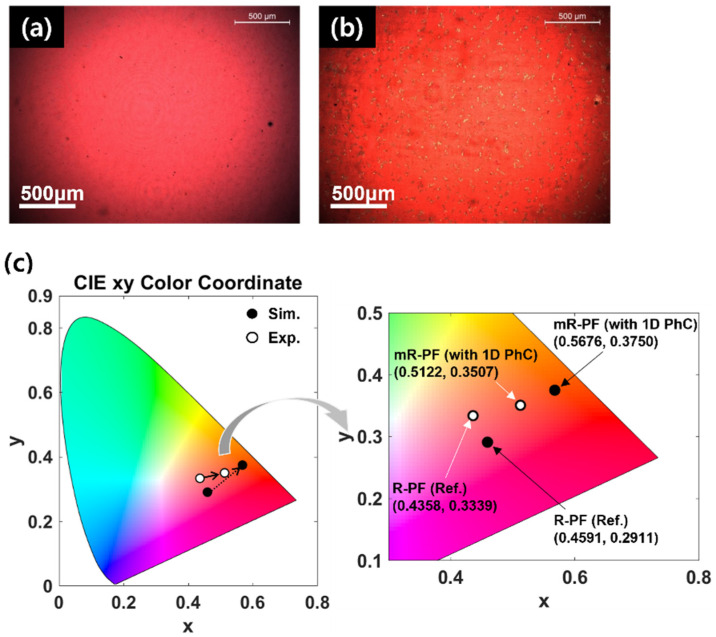
Optical characteristics of the fabricated and simulated PCFs. The microscopic image of the fabricated (**a**) R-PF and (**b**) mR-PF, respectively. (**c**) CIE chromaticity diagram.

Figure 6 shows the optical characteristics of the fabricated and simulated filters. For quantitative analysis, the transmittance spectrum of each filter is mapped to the CIE 1931 color coordinate, as shown in Figure 6c. The coordinates in the CIE 1931 color coordinate are (0.4358, 0.3339) (Exp_R-PF), (0.5122, 0.3507) (Exp_mR-PF), (0.4591, 0.2911) (Sim_R-PF), and (0.5676, 0.375) (Sim_mR-PF). The proposed structures confirm that by adding the 1D PhC structure, the color purity is enhanced by moving the color coordinate to the outside point (0.078) (Exp.) and (0.137) (Sim.).

As a result of adding the PhC to the PCF, undesired transmission in the low wavelength region decreased, resulting in better color purity. As shown in Figure 3, the PCF and PhC in mR-PF can be analyzed separately. To minimize new or lost optical modes between PCF and PhC, the LiF was used as the first layer of PhC. It can also eliminate one step of the PhC deposition process. It can be used for multilayer structures of organic light-emitting diodes made by thermal evaporation or plasma-enhanced chemical vapor deposition, such as thin-film encapsulation or distributed Bragg reflectors comprising materials with a high refractive index ratio.

## 4. Conclusions

We demonstrate a novel PCF with a 1D PhC structure, which exhibits a distinguished improvement in color purity with only a few additional layers. We performed an optical simulation to investigate the mechanism and transmittance properties of the mR-PF. By the PBG of the 1D PhC, the undesirable transmission peaks in the low wavelength region were suppressed, and the bandwidth and transmittance were adjustable. These results enhanced the filtering characteristic, particularly the selectivity of the pass-band. The 1D PhC in the mR-RF influences the variation in the SP_top_ resonant mode. When the electric field is concentrated on the upper or lower Al and LiF interfaces in the second-order resonant mode, the electric field in the z-axis direction is reduced by the SP penetration depth and does not contribute to the transmissive energy. It causes an inflection point in the mR-PF transmissive spectrum. In addition, the suggested simple structure is advantageous for mass production because the multilayer, which forms a PhC structure, does not require any complex patterning technologies. We expect that good color purity and simple structure render PCFs favorable as alternatives to the conventional color filters in industrial devices.

## Data Availability

The data presented in this study are available on request from the corresponding author.

## Supplementary Materials

The following supporting information can be downloaded at: https://www.mdpi.com/article/10.3390/nano12101694/s1, Figure S1: Transmission spectra of LiF/WO_3_ pairs.

## Appendix A

We examine the spectral response of the multilayers according to the number of repetitions for optimizing with the smallest numbers of pairs. As shown in Figure S1, the valley of transmittance is shaped asymmetrically as broadening on the longer wavelength side. In this regard, $\lambda_{PBG\_C}$ was set to 445 nm, not 450 nm corresponding to *λ_sp(1,1)_*. From Equation (2), the thickness of the WO_3_ and LiF was set to 53 nm and 80 nm, respectively. As explained earlier, most spectra have a valley near 445 nm and shallow ripples on the longer wavelength region. Within the visible light range, the valley around $\lambda_{PBG\_C}\;$is distinguishable when the pairs of the WO_3_ and LiF were stacked at least 3 times.

Meanwhile, the more layers are stacked, the stronger effect of the PBG is performed and finally the deeper and the narrower the valley of $\lambda_{PBG}$ is. Therefore, the peak exhibits a blue shift with additional layers. It appears at 692, 605, and 572 nm with 3, 4, and 5 pairs of the LiF and WO_3_, respectively. Additionally, accumulated interfaces exhibit multiple interference modes between reflected lights from each boundary, so increases in the denser spectral occurrence of peaks in the ripple. When the number of stacks is larger than 3 pairs, shallow valleys appear in the wavelength range from 630 nm to 730 nm: 710 nm in case of 4 pairs; 642 nm in case of 5 pairs.

The case of the 3 pairs of the LiF and WO_3_ is suitable for the purpose of enhancing color purity of the R-PF. Its transmission spectrum shows a broad valley covering the range from 400 nm to 600 nm which is an undesired transmission of the R-PF with a minimum transmittance of 25.3%. Additionally, it has a high transmittance at the maximum peak wavelength region of the R-PF (634 nm).
