# Supplementary figures and images for: High Color Purity Plasmonic Color Filter by One-Dimensional Photonic Crystals
Source: Nanomaterials (Basel). 2022 May 16;12(10):1694. doi: 10.3390/nano12101694 (PMC9145173; doi:10.3390/nano12101694)

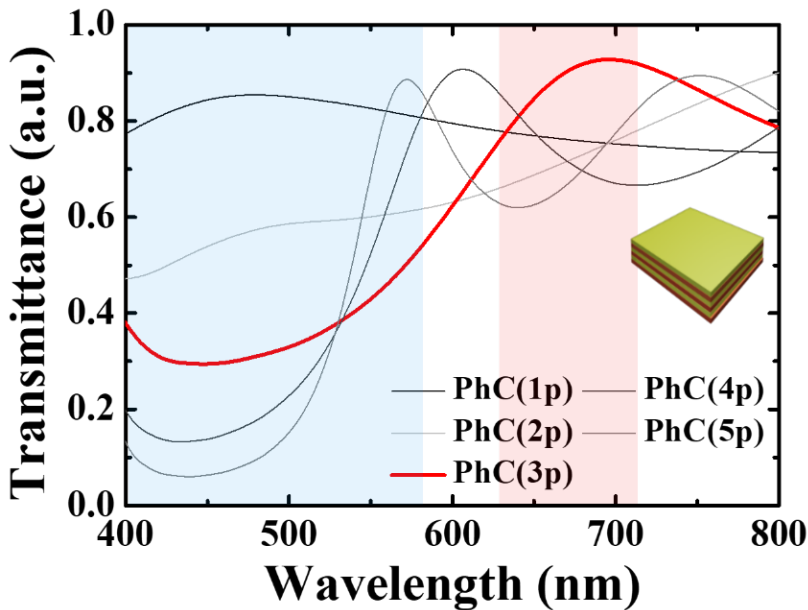

Figure S1. Transmission spectra of LiF/ WO<sub>3</sub> pairs.

Supplement: Supplementary file 1 [file nanomaterials-12-01694-s001.zip › nanomaterials-1669541-supplementary.pdf]
